# Hyoscyamine in Spasmodic and Convulsive Neurosis, Etc.

**Published:** 1873-07

**Authors:** 


Hyoscyamine in Spasmodic and Convulsive Neurosis, Mercurial and Senile Tremor, Tetanus, etc.—The last number of the *Bulletin Generale Therapeutique*, (Dec. 15, '72,) leads off with a long article upon this subject. The following are the conclusions arrived at:

1. Hyoscyamine represents all the active principles of the drug from which it is derived. Its fixidity of composition gives to the results obtained under its use a precision that can not be secured by the drug itself.

2. Hyoscyamine should be administered at first in light doses (2 milligrammes a day) either in pill or in hypodermic injection. The dose may then be gradually increased to ten or even twelve milligrammes per day.

3. It should be continued, even though slight symptoms of toxic action present, as dryness of the throat, dilatation of the pupils, etc. But if these symptoms become grave, it should be suspended. These symptoms are fugitive, and disappear in the course of a few hours.

4. Hyoscyamine exercises on man a narcotic action. It is efficacious against pain and neuralgias in particular; but its efficacy is less than that of opium and belladonna.

5. This medicament exercises a favorable action in spasmodic and convulsive neuroses. It cures mercurial tremor even after every other remedy has proven futile. It produces a marked amelioration in paralysis agitans.

6. Its action is null in locomotor axtaxia. In traumatic tetanus, although the patient died, it procured a decided remission in the symptoms, but the question of its use in this affection may not yet be regarded as settled, as new observations are required.—*The Clinic.*

